# Large-Cell Neuroendocrine Carcinoma of the Ampulla of Vater: A Systematic Literature Review of a Rare and Aggressive Entity

**DOI:** 10.7759/cureus.109206

**Published:** 2026-05-19

**Authors:** Evangelia Florou, Archana Abarnadevi Karthikeyan, Shirin Ubaid, Raj Srirajaskanthan, Parthi Srinivasan, Andreas Prachalias

**Affiliations:** 1 Hepato-Pancreato-Biliary Surgery, King's College Hospital, London, GBR; 2 Gastroenterology, King's Health Partners Neuroendocrine Tumour Centre, King's College Hospital, London, GBR; 3 Hepato-Pancreato-Biliary Surgery and Liver Transplantation, London Bridge Hospital, London, GBR

**Keywords:** ampulla of vater, ampullary adenocarcinoma, large-cell neuroendocrine carcinoma, neuroendocrine neoplasm, periampullary tumour, small-cell neuroendocrine carcinoma, systematic review

## Abstract

Neuroendocrine carcinomas of the ampulla of Vater are rare but highly aggressive, poorly differentiated neoplasms associated with poor outcomes. This contrasts with ampullary adenocarcinoma, which is generally associated with more favourable survival following curative resection, and with well-differentiated periampullary neuroendocrine tumours, which typically demonstrate more indolent behaviour and better prognosis.

A Preferred Reporting Items for Systematic Reviews and Meta-Analyses (PRISMA)-compliant systematic review was conducted across PubMed/MEDLINE, Embase, Scopus, Web of Science, and Google Scholar (from inception to July 2025) to identify reported cases of large-cell neuroendocrine carcinoma (LCNEC) of the ampulla of Vater. Studies reporting extractable patient-level clinicopathological, treatment, and outcome data were included. Data were standardised and synthesised descriptively due to the rarity and heterogeneity of the condition.

A total of 22 patient-level cases of LCNEC of the ampulla of Vater were identified from 14 studies. The median age was 70 years (range: 44-84), with a male predominance. Tumours were generally small at presentation (median size approximately 20 mm); however, lymph node metastasis was present in 16 of 20 evaluable patients (80%). All patients underwent pancreaticoduodenectomy or pylorus-preserving pancreaticoduodenectomy. Adjuvant chemotherapy was administered in 12 of 22 patients (55%), most commonly platinum-based regimens. Tumour recurrence occurred in approximately 55-60% of cases, most frequently involving the liver. Median overall survival was approximately 11 months, with eight patients (36%) alive at the last follow-up.

LCNEC of the ampulla of Vater is an aggressive malignancy with poor prognosis despite surgical resection. This review highlights a clinically important “size-biology paradox,” whereby relatively small tumours demonstrate early metastatic behaviour and adverse outcomes. Tumour biology appears to outweigh conventional staging parameters, underscoring the need for early recognition, accurate histopathological classification, and consideration of systemic therapy. Further multicentre studies are required to optimise management strategies and improve patient outcomes.

## Introduction and background

Ampullary carcinoma is a malignancy arising from the ampulla of Vater and is generally associated with a more favourable prognosis compared with pancreatic or distal bile duct cancers. This is largely attributed to earlier symptom onset, most commonly obstructive jaundice, allowing for timely diagnosis and higher rates of resectability. Reported five-year overall survival following curative resection ranges between 40% and 50%, with outcomes influenced by tumour stage, nodal status, and histological subtype [1-3].

Neuroendocrine neoplasms of the periampullary region are uncommon and comprise a heterogeneous group of tumours, including well-differentiated neuroendocrine tumours (NETs) and poorly differentiated neuroendocrine carcinomas (NECs). Well-differentiated NETs are generally associated with favourable outcomes, with reported five-year survival rates ranging from 60% to 90% [4]. In contrast, NECs are high-grade malignancies characterised by aggressive clinical behaviour and significantly worse prognosis, with reported five-year survival rates of approximately 10-15% [5].

According to the 2019 World Health Organization (WHO) Classification of Tumours of the Digestive System, NECs are subdivided into small-cell and large-cell subtypes, both classified as grade 3 (G3) neoplasms [6]. Large-cell neuroendocrine carcinoma (LCNEC) is defined histologically by large polygonal cells with abundant cytoplasm, prominent nucleoli, high mitotic activity, and extensive necrosis [6-9]. These tumours typically demonstrate a high proliferative index (Ki-67 >20%), often exceeding 50%, which is associated with early metastatic potential and aggressive disease course [10-12].

LCNEC of the ampulla of Vater represents an exceptionally rare entity within this spectrum [7,8,12]. Reported cases remain geographically diverse but extremely limited, with available evidence consisting primarily of isolated case reports and small series from Asia, Europe, and North America [7,8,12]. Patients most commonly present with obstructive jaundice and nonspecific biliary symptoms related to biliary obstruction [5,12].

The pathogenesis of ampullary LCNEC remains poorly understood; however, proposed mechanisms include dedifferentiation from pre-existing adenocarcinoma or transformation from pluripotent epithelial stem cells capable of neuroendocrine differentiation [5,12].

Diagnosis requires recognition of characteristic large-cell morphology in conjunction with neuroendocrine differentiation confirmed by immunohistochemical markers such as chromogranin A, synaptophysin, and CD56 [6,7]. However, overlap with adenocarcinoma and mixed neuroendocrine-non-neuroendocrine neoplasms (MiNENs) may pose diagnostic challenges, often necessitating comprehensive histopathological and multidisciplinary evaluation [8,12].

Given its rarity, the available evidence on ampullary LCNEC is limited to isolated case reports and small case series, resulting in a lack of standardised management strategies and limited understanding of its clinical behaviour. To our knowledge, this is the first Preferred Reporting Items for Systematic Reviews and Meta-Analyses (PRISMA)-compliant systematic review focusing on patient-level outcomes and prognostic behaviour of ampullary LCNEC.

## Review

Materials and methods

This systematic review was conducted in accordance with the PRISMA 2020 guidelines [13]. A comprehensive literature search was performed in PubMed/MEDLINE, Embase, Scopus, Web of Science, and Google Scholar from database inception to July 2025. Search terms included combinations of keywords and Medical Subject Headings (MeSH), including “large-cell neuroendocrine carcinoma,” “LCNEC,” “neuroendocrine carcinoma,” “ampulla of Vater,” and “ampullary.” Reference lists of included studies were manually screened to identify additional relevant articles.

The initial search yielded 362 records, of which 224 remained after duplicate removal. Following title and abstract screening, 40 full-text articles were assessed for eligibility. Of these, 26 were excluded; 12 articles did not represent LCNEC histology, six articles were non-ampullary primaries, and eight articles lacked extractable patient-level data. Fourteen studies were included in the final analysis. The study selection process is illustrated in a PRISMA flow diagram.

Eligible studies included case reports and case series describing LCNEC of the ampulla of Vater with extractable patient-level clinicopathological, treatment, and outcome data. Studies reporting mixed neoplasms were included only when the LCNEC component was predominant or separately analysable. Exclusion criteria comprised well-differentiated NET (G1-G2), small-cell NEC, non-ampullary primaries, review articles without individual patient data, conference abstracts without full reports, and guideline or staging publications. Study selection was performed independently by two reviewers, with discrepancies resolved by consensus.

Individual patient-level data were extracted wherever possible. For studies reporting multiple patients, individual cases were extracted separately when sufficient clinicopathological information was available. Extracted variables included demographics, tumour characteristics, staging, histology, treatment, recurrence, and survival outcomes.

To enable pooled descriptive analysis, variables were standardised across studies. Histologic classification was harmonised into three categories: pure LCNEC, predominant LCNEC with focal glandular differentiation, and MiNENs. In studies reporting combined high-grade NECs, only cases explicitly identified as LCNEC were included. Surgical procedures were categorised as pancreaticoduodenectomy or pylorus-preserving pancreaticoduodenectomy, and resection margins were classified as R0, R1, or not reported (NR, when margin status was unavailable in the original publication). Adjuvant therapies were grouped into broader categories (e.g., platinum-based regimens) due to heterogeneity in chemotherapy protocols and incomplete reporting across studies.

Lymph node involvement was derived from reported nodal staging when not explicitly stated, and recurrence was recorded as present or absent. Tumour size was recorded only when explicitly reported and was not inferred from TNM staging. Missing variables were recorded as NR and were not imputed. For patients alive at the last follow-up, overall survival was defined as the duration of follow-up.

**Figure 1:**
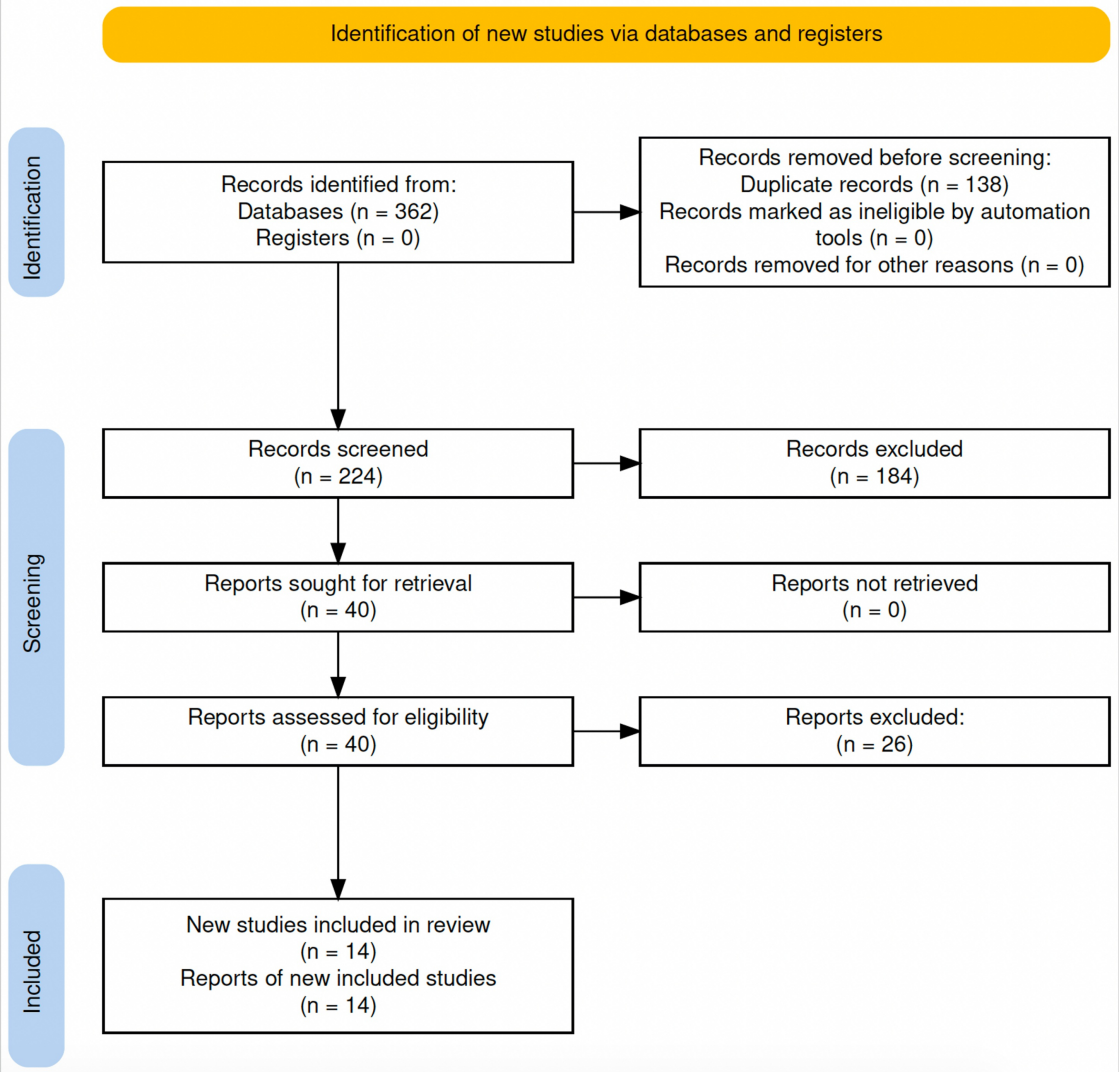
PRISMA flow diagram of the study process. Flow diagram illustrating the identification, screening, eligibility assessment, and inclusion of studies in this systematic review, in accordance with PRISMA 2020 guidelines. Reasons for full-text exclusion included non-LCNEC histology, non-ampullary primary tumours, and lack of extractable patient-level data. PRISMA: Preferred Reporting Items for Systematic Reviews and Meta-Analyses; LCNEC: large-cell neuroendocrine carcinoma

Series-level data without extractable patient-level information were excluded from quantitative analysis but reviewed qualitatively. Given the rarity and heterogeneity of LCNEC, findings were synthesised descriptively without formal meta-analysis. A formal risk of bias assessment was not performed due to the inclusion of case reports and small case series. This review was not prospectively registered. The study selection process is summarised in Figure 1.

Results

A total of 22 published patient-level cases of LCNEC of the ampulla of Vater were identified from 14 studies. The median age was 70 years (range: 44-84), with a male predominance (59%). Obstructive jaundice was the most commonly reported presenting symptom and was documented in 11 of 13 evaluable patients (85%), although symptom reporting was incomplete in several cases, particularly in the series by Nassar et al. [5].

All patients underwent pancreaticoduodenectomy or pylorus-preserving pancreaticoduodenectomy, reflecting standard surgical management.

Histologically, pure LCNEC accounted for 14 cases (64%), while mixed LCNEC (MiNEN) was identified in eight cases (36%). Tumours were generally small, with a median size of approximately 20 mm (range: 10-31 mm). Despite the small tumour size, lymph node metastasis was present in 16 of 20 evaluable patients (80%), indicating early dissemination.

Adjuvant chemotherapy was administered in 12 patients (55%), most commonly platinum-based regimens. During follow-up, tumour recurrence occurred in approximately 12of 22 patients (55%), most frequently involving the liver.

Survival outcomes were poor, with a median overall survival of approximately 11 months. Only eight patients (36%) were alive at the last follow-up, including a small number of long-term survivors. Most deaths occurred within the first 12 months following diagnosis or surgical resection, reflecting the highly aggressive biological behaviour of LCNEC despite curative-intent treatment.

A summary of all reported cases of LCNEC of the ampulla of Vater is shown in Table 1. Variables include demographics, tumour characteristics, treatment, and outcomes.

**Table 1:** Summary of patient-level clinicopathological characteristics of ampullary LCNEC. LCNEC: large-cell neuroendocrine carcinoma; NR: not reported; PD: pancreaticoduodenectomy; PPPD: pylorus-preserving pancreaticoduodenectomy; OS: overall survival; AWD: alive with disease

Patient ID	Age	Sex	Jaundice	Size (mm)	T stage	N stage	M stage	Histology	Ki-67 (%)	Surgery	Margin	Adjuvant chemo	Recurrence	Site of recurrence	OS (months)	Status
Cavazza et al. [7]	74	F	Yes	30	T2	N1	M0	Pure	NR	PD	R0	No	Yes	Liver, bone	8	Dead
Stojsic et al. [8]	60	M	Yes	30	T2	N1	M0	Pure	41	PD	R0	Yes	Yes	Liver	11	AWD
Hartel et al. [12]	44	F	Yes	16	T2	N1	M0	Pure	NR	PPPD	R0	NR	NR	NR	NR	NR
Cheng et al. [14]	55	F	No	18	T1	N1	M0	Mixed	60	PD	R0	No	Yes	Liver, peritoneum	6	Dead
Huang et al. [15]	59	M	Yes	28	T1b	N1	M0	Pure	NR	PD	NR	Yes	Yes	Liver	10	Dead
Sunose et al. [16]	73	F	Yes	25	T3	NR	NR	Mixed	NR	PD	NR	Yes	Yes	Liver, bone	13	Dead
Imamura et al. [17]	81	M	No	14	T3	N0	NR	Pure	89	PPPD	NR	No	Yes	Liver	11	Dead
Imamura et al. [17]	72	M	NR	24	T3	N1	NR	Mixed	67	PPPD	NR	Yes	No	—	24	Alive
Sonmez et al. [18]	78	M	Yes	15	NR	N0	NR	Pure	80	PPPD	R0	No	Yes	Liver	NR	Dead
Karlafti et al. [11]	70	M	Yes	31	T3	N1	M0	Pure	45	PPPD	R0	Yes	NR	NR	NR	NR
Kagaya et al. [19]	70	F	Yes	18	T2	NR	NR	Pure	>90	PD	R0	Yes	Yes	NR	74	Alive
Nassar et al. [5]	61	M	NR	NR	NR	N1	M0	Mixed	NR	PD	NR	Yes	Yes	NR	6	Dead
Nassar et al. [5]	75	M	NR	NR	NR	N1	M0	Pure	NR	PD	NR	No	No	—	24	Alive
Nassar et al. [5]	84	M	NR	NR	NR	N1	M0	Mixed	NR	PD	NR	Yes	Yes	NR	8	Dead
Nassar et al. [5]	50	F	NR	NR	NR	N1	M0	Pure	NR	PD	NR	Yes	Yes	NR	10	Dead
Nassar et al. [5]	77	M	NR	NR	T3	N1	M0	Mixed	NR	PD	NR	No	No	—	36	Alive
Nassar et al. [5]	80	M	NR	NR	NR	N1	M0	Pure	NR	PD	NR	Yes	Yes	NR	5	Dead
Nassar et al. [5]	55	M	NR	NR	NR	N1	M0	Pure	NR	PD	NR	Yes	Yes	NR	7	Dead
Nassar et al. [5]	68	F	NR	NR	NR	N1	M0	Pure	NR	PD	NR	No	No	—	18	Alive
Liu et al. [20]	70	F	Yes	10	T2	N0	M0	Pure	>90	PD	R0	No	No	—	1	Alive
Zhang et al. [21]	69	M	Yes	15	T2	N0	M0	Mixed	70	PD	NR	No	No	—	33	Alive
Florou et al. [22]	69	F	Yes	16	T2	N1	M0	Mixed	70	PD	R0	Yes	No	—	8	Alive

Discussion

NECs of the ampulla of Vater represent a rare but highly aggressive subset of periampullary malignancies, with outcomes significantly worse than those observed in conventional ampullary adenocarcinoma. While ampullary adenocarcinoma is typically associated with earlier presentation, higher resectability, and reported five-year survival rates of approximately 40-50%, prognosis in NECs remains poor despite similar surgical management [1-3]. This disparity is largely attributable to the biological behaviour of NECs, which are characterised by high proliferative activity, early lymphovascular invasion, and a strong propensity for nodal and distant metastasis. Prior studies have demonstrated that poorly differentiated NECs exhibit rapid disease progression, with five-year survival rates as low as 10-15% [5,23]. Even following curative-intent pancreaticoduodenectomy, recurrence is frequent, underscoring the aggressive nature of these tumours compared with adenocarcinoma.

Small-cell neuroendocrine carcinoma (SCNEC) represents the most aggressive end of the NEC spectrum. Although ampullary SCNEC is exceedingly rare, available evidence consistently demonstrates rapid disease progression, early systemic dissemination, and poor survival outcomes [5,23]. Median overall survival is typically reported in the range of 6-12 months, with five-year survival rarely exceeding 10% [5,23]. Similar to LCNEC, SCNEC frequently presents with nodal and distant metastases at diagnosis; however, its biological behaviour and chemosensitivity patterns more closely resemble small-cell carcinoma of pulmonary origin [10,23,24]. Despite the use of platinum-based chemotherapy, outcomes remain poor, further emphasising the aggressive biology of high-grade NECs [22].

Within this spectrum, LCNEC of the ampulla of Vater also demonstrates highly aggressive behaviour. Published case-level evidence, including the present PRISMA-compliant review, shows that LCNEC is characterised by early recurrence and poor survival despite curative-intent resection. In this study, tumours were frequently small at presentation (median size approximately 20 mm), yet lymph node metastasis was present in the majority of patients, and recurrence occurred in over half of cases. Median overall survival remained limited at approximately 11 months, consistent with previously reported outcomes in the literature [6,7,13-22]. These findings confirm that LCNEC behaves more aggressively than both ampullary adenocarcinoma and well-differentiated periampullary NETs.

A key finding of this study is the apparent dissociation between the tumour size and biological behaviour in LCNEC. In contrast to ampullary adenocarcinoma, where prognosis closely correlates with the tumour stage and nodal involvement, LCNEC demonstrates a markedly different pattern. In adenocarcinoma, patients with early-stage, node-negative disease (T1-T2 N0) may achieve five-year survival rates of 70-80%, whereas nodal involvement reduces survival to approximately 25-40% [1-3]. Tumour size in this setting is generally associated with disease stage and prognosis. However, in LCNEC, relatively small tumours frequently exhibit early lymph node metastasis and aggressive clinical behaviour. This “size-biology paradox” highlights that tumour size alone is an unreliable prognostic marker in LCNEC, with tumour biology appearing to outweigh conventional anatomical staging.

Previous narrative reviews have described ampullary LCNEC [11]. However, these lacked systematic methodology and detailed patient-level outcome analysis. The available literature remains limited to isolated case reports and small series [7,8,11,12,14-22], with inconsistent reporting of outcomes and treatment strategies, which hampers meaningful prognostic interpretation.

The present study is subject to several limitations, including the retrospective nature of the included reports, small sample size, and heterogeneity in data reporting. Furthermore, reliance on case reports introduces potential publication bias. Several clinically important variables, including presenting symptoms, Ki-67 index, margin status, adjuvant therapy details, recurrence patterns, and long-term survival outcomes, were inconsistently reported across studies, limiting the ability to perform subgroup analyses and comparative outcome assessment.

Collectively, these findings suggest that LCNEC may represent one of the most aggressive primary malignancies of the ampulla of Vater, with prognosis driven predominantly by tumour biology rather than conventional clinicopathological parameters. These findings support consideration of LCNEC as a biologically distinct entity requiring tailored multidisciplinary management. Future studies should incorporate standardised clinical evaluation, detailed histopathological characterisation, and molecular/genetic profiling to better define prognostic factors and therapeutic targets in ampullary LCNEC.

## Conclusions

LCNEC of the ampulla of Vater is an exceptionally rare but highly aggressive malignancy associated with early nodal and distant metastatic dissemination, frequent recurrence, and poor survival despite curative-intent surgical resection. Findings from this systematic review highlight a clinically important “size-biology paradox,” whereby relatively small tumours may already demonstrate highly aggressive behaviour and adverse outcomes.

Prognosis appears to be driven predominantly by tumour biology rather than conventional staging parameters. Early recognition, accurate histopathological classification, and multidisciplinary management are essential. Given the rarity of this entity, continued reporting of additional cases and multicentre collaborative studies will be important to improve understanding and optimise management strategies.
